# Short and medium-term effects of an education self-management program for individuals with osteoarthritis of the knee, designed and delivered by health professionals: A quality assurance study

**DOI:** 10.1186/1471-2474-9-117

**Published:** 2008-09-08

**Authors:** Sophie Coleman, Kathryn Briffa, Heather Conroy, Richard Prince, Graeme Carroll, Jean McQuade

**Affiliations:** 1 Arthritis Foundation of Western Australia, PO Box 34, Wembley, Western Australia, 6913, Australia; 2 Department of Physiotherapy, Curtin University of Technology, Bentley, Western Australia, 6102, Australia; 3 Department of Medicine, University of Western Australia, Nedlands, Western Australia, 6009, Australia; 4 Cnr Park & Guildford Rds, Mount Lawley, Western Australia, 6055, Australia

## Abstract

**Background:**

Self-management (SM) programs are effective for some chronic conditions, however the evidence for arthritis SM is inconclusive. The aim of this case series project was to determine whether a newly developed specific self-management program for people with osteoarthritis of the knee (OAK), implemented by health professionals could achieve and maintain clinically meaningful improvements.

**Methods:**

*Participants: *79 participants enrolled; mean age 66, with established osteoarthritis of the knee. People with coexisting inflammatory joint disease or serious co-morbidities were excluded.

*Intervention: *6-week disease (OA) and site (knee) specific self-management education program that included disease education, exercise advice, information on healthy lifestyle and relevant information within the constructs of self-management. This program was conducted in a community health care setting and was delivered by health professionals thereby utilising their knowledge and expertise.

*Measurements: *Pain, physical function and mental health scales were assessed at baseline, 8 weeks, 6 and 12 months using WOMAC and SF-36 questionnaires. Changes in pain during the 8-week intervention phase were monitored with VAS.

**Results:**

Pain improved during the intervention phase: mean (95% CI) change 15 (8 to 22) mm. Improvements (0.3 to 0.5 standard deviation units) in indices of pain, mental health and physical functioning, assessed by SF-36 and WOMAC questionnaires were demonstrated from baseline to 12 months.

**Conclusion:**

This disease and site-specific self-management education program improved health status of people with osteoarthritis of the knee in the short and medium term.

## Background

With an ageing population, the prevalence of chronic disease is increasing. Osteoarthritis of the knee is a widespread chronic condition and one of the most common causes of musculoskeletal disability [1]. Complementary to conventional medical care, self-management interventions are considered to be beneficial in the management of people with chronic illness [2,3]. These interventions are designed to assist people to effectively manage their condition (between physicians visits), by teaching them how to cope with their symptoms, including the physical and psychological consequences of living with a chronic disease.

Approaches to self-management vary [4]. The majority of self-management interventions are led by health professionals (HP) in a group setting where all participants are affected by the same condition. Health professionals are a credible source of information for participants and have the knowledge to provide factual disease-specific education and respond to queries where required. When all members of a group have the same condition, all components of the intervention can be tailored to the specific needs of the group.

Notable exceptions to this approach are the Chronic Diseases and Arthritis Self-Management Programs (ASMP) developed at Stanford University [2,5]. These programs are also delivered in a group setting but are led by trained lay tutors. They have a more generic approach as they are catering for participants with a variety of different conditions in the one group. This approach is cheaper to deliver but cost-effectiveness is yet to be established [6,7]. Participants in the arthritis groups may include people with a variety of different rheumatic diseases.

The Arthritis Self-Management Program has been tested widely with the majority of studies conducted in the USA or UK. Many, but not all of these studies have found the program to be effective. Overall, Warsi et al (2004), in their systematic review of self-management interventions for various chronic diseases, found a trend towards a small benefit from arthritis programs, the majority being ASMP or ASMP derivatives, but the results were not significant and there was suggestion of publication bias [4].

In view of the high prevalence of OA knee in the community, we considered the development of a specific program to be justified. The goals of the program were to reduce pain, improve physical function and increase general well being. The program was designed to be delivered by HP's including physiotherapists, nurses and occupational therapists. It included disease specific education, including precise information on medications and analgesia as well as the importance of exercise and weight management. A social cognitive theory approach incorporating goal setting, problem solving and cognitive techniques was adopted to improve self-efficacy and facilitate long-term change in behaviour. Participants were encouraged to include exercise and effective pain management as well as specific information learned during the sessions in their weekly goal setting. This approach is a means of encouraging participants to incorporate specific education learned from week to week relevant to their disease [8,9]. The HP leaders can provide support and specific feedback for any problems that were encountered.

The newly developed OAK program was implemented as a clinical service of the Arthritis Foundation of Western Australia (WA). We report here the progress of participants during the implementation phase and 12 month follow up. The aim was to determine whether participants had experienced improvements in quality of life, pain, stiffness, and physical function, and whether these improvements would be maintained for 12 months.

## Methods

This case series quality assurance project was conducted within the clinical services provided at Arthritis Western Australia. Public awareness of the programs offered by Arthritis WA is often generated via General Practitioner referral or suggestion from friends or family. Programs and services are also advertised in community newspapers; quarterly Arthritis WA magazines; and local radio stations, often linked to health or scientific articles.

This quality assurance project was given Institutional approval by the Board of Arthritis WA and the OA Knee Advisory Committee and complies with National Health and Medical Research Council (Australia) criteria for quality assurance programs [10].

### Participants

People with OA knee enquiring consecutively to Arthritis WA about access to appropriate services were invited to participate in the new disease specific self-management program. Those who were not interested in the OAK program, did not meet the selection criteria, or were not confident they would be able to participate fully were encouraged to utilize other appropriate services of Arthritis WA.

Only those with a diagnosis of OA of the knee were enrolled. It was a requirement that the diagnosis was confirmed by the participant's medical practitioner. Diagnostic criteria were at the discretion of the doctor. Disease severity was not a selection criterion. Unilateral total knee replacement did not preclude enrolment. Other criteria for ineligibility was age greater than 85 years; inability to walk 300 meters; inflammatory joint disease including rheumatoid arthritis; major concurrent illness such as cancer; bilateral knee replacement; knee surgery scheduled within 6 months of commencing the program; or physical impairments that precluded fulfilling the requirements of the program. Those people were referred to other available services.

This project was consistent with the National Health and Medical Research of Australia definition for a quality assurance project [11]. The OAK program and the associated clinical assessments were clearly described to all volunteers who had the opportunity to have all questions answered and provided verbal consent to participate.

### Intervention

Groups of 8–10 participants attended 6 education sessions (one 2.5-hour session per week). Participants were provided with written material relevant to the information component discussed each week. The program used a holistic approach, including a range of aspects of care such as:

• Pain management strategies

• Exercise advice

• Joint protection

• Medication/analgesia

• Balance and falls prevention

• Coping with negative emotions

• Fatigue

• Self-management skills (goal setting, problem solving, cognitive techniques)

The fidelity of the OAK program was maintained by the use of a facilitator's manual with modules for program delivery each week. The program was delivered by 2 nurses and assessments were performed by 3 physiotherapists who had no contact with the participants other than during the assessment sessions. Participants were assessed by the same physiotherapist whenever possible to ensure consistency. The assessors did not participate in the facilitation of the program. It was a requirement that health professionals who delivered the program meet minimum musculoskeletal knowledge requirements.

Attendance was recorded at each of the 6 intervention sessions and at each assessment time-point.

### Response to Intervention

Participants were assessed at baseline, immediately post-intervention at 8 weeks, and at 6 and 12 months after the program. In addition, pain was assessed on a week-to-week basis during the first 8 weeks using a VAS.

### Assessments included

*Health status *was assessed using both a disease specific and a generic index as follows:

• The disease specific WOMAC Osteoarthritis Index (WOMAC LK3.0) assesses pain, stiffness and physical function in people with OA of the hip or knee [12]. Validity and reliability of the WOMAC pain, physical function and stiffness subscales are well established and the questionnaire is sufficiently sensitive to detect changes in health status in response to intervention [12].

• The generic Medical Outcomes Study Short Form 36 Version 1 questionnaire (SF-36). The SF-36 was designed to provide a profile of scores that would be useful in understanding the health burden in chronic diseases and the effect of treatment on general health status. It includes 8 component sub-scales that correspond to aspects of physical and mental health and well being [13]. Adequate reliability for between group comparisons has been demonstrated in numerous studies and an English version of the questionnaire has been developed and validated specifically for use in Australia [14]. For people with OA, an improvement of 5 points on the physical component score of the SF-36 is considered to be clinically significant [15].

**Figure 1:**
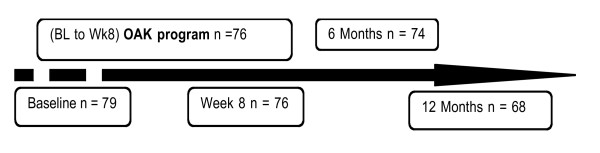
**Flow chart time-points with number of participants attending**. Baseline to week 8 (intervention), 6 months and 12 months (follow-up assessments)

*Pain *was assessed using pain scales included in the WOMAC and SF-36 indices. During the intervention period, pain was monitored on a week-to-week basis (Figure 1) using a 100 mm VAS. The left hand anchor was "No Pain", and the right hand anchor "Worst Pain". The VAS is well established in clinical practice for measuring pain levels post-surgery, following drug therapy and other interventions in arthritis populations [16]. A reduction of 30% or 2 points in VAS is considered to represent a clinically important difference [17,18].

*Active range of motion *of knee flexion and extension were measured using a long armed goniometer [19]. The reliability and validity of the goniometer is well established for measurement of active knee flexion and extension [19,20].

*Balance *was assessed using a timed single leg balance test. This simple test assesses the length of time, to a maximum of 10 seconds, a person can stand on one leg. It is a good predictor of falls in the elderly [21] and is reliable and valid [22].

### Statistical Analysis

Data were analysed using SPSS v16 for Macintosh. One-way (repeated measures) analysis of variance with *time *(baseline, 8 weeks, 6 months and 12 months) as the independent variable was used to assess changes in the variables of interest. Where the ANOVA was significant pair-wise differences between baseline and 12 months were compared using paired t-tests and mean change and 95% confidence interval for were calculated. The effect size for the pair wise comparison was calculated using Cohen's *d *(the difference between the means: M_1 _- M_2 _divided by the standard deviation). Improvement is represented as a positive difference: small, *d *= 0.2; medium *d *= 0.5; large *d *= 0.8 [23] allowing the comparison of outcomes across the intervention. Separate models were constructed for each outcome variable. Normal distribution and homogeneity of the variance were confirmed prior to further analysis. Statistical significance was inferred at a 2-tailed p < 0.05.

## Results

141 people expressed interest in the OAK program. Recruitment for the project was discontinued when 8 groups of eligible participants (19 men, 60 women, mean (SD) age 66 (9) had enrolled in the program. Of these, 68 participants completed the program and returned for all the follow-up assessments to 12 months. Those who were absent or likely to be absent for more than 2 of the 6 sessions were deferred to the next group (Table 1). All the participants included in the analyses attended at least 4 of the 6 self-management sessions with the average attendance being 5.8 sessions. The reasons cited for withdrawal were overseas relocation and work, family, and time commitments. Close to 90% of participants had other co-existing disease (Table 1).

**Table 1:** Characteristics of participants enrolled in OAK program

**Gender (F:M)**	**60:19**
**Age: mean (SD) years**	**66 (9)**
**Socio-Economic Index by Post Code **[24]	**Number (%)**

Index measured in quintile ranges	
Top 25%	59 (74.6)
50–75%	7 (8.8)
25–50%	7 (8.8)
10–25%	3 (3.8)
Bottom 10%	3 (3.8)

**Coexisting disease**	n (%)*

Cardiovascular, n (%)	48 (45)
Mental Health, n (%)	9 (11)
Gastrointestinal, n (%)	27 (30)
Endocrine, n (%)	15 (18)
Musculoskeletal, n (%)	16 (20)
Osteoporosis, n (%)	14 (18)
Multiple co-morbidities, n (%)	51 (64.5)
Other, n (%)	31 (61)
No co-morbidities, n (%)	12 (15)

*Percentage adds > 100 as some participants have more than one coexisting disease

Socio-economic status was estimated according to residential address postcodes using a method developed by the Australian Bureau of Statistics – "The Index of Relative Socio-Economic Disadvantage" [24]. This index provides a weighted value that includes variables that reflect or measure disadvantage. These variables include: low-income, low educational attainment, high unemployment, and low skilled occupations. A low index value represents disadvantage and a high index value represents advantage in an area. Participants in the OAK program were over represented in the highest group (Table 1).

There was a significant improvement (*p *< 0.05) in each of the dimensions of health status (pain, stiffness, physical function and total) measured by the WOMAC questionnaire (Table 2). These improvements were evident by the completion of the intervention phase and were maintained until the 12 month follow-up (Table 2). There were also significant improvements in all of the 8 quality of life domains measured using the SF-36 (Table 3).

**Table 2:** WOMAC scores at baseline, 8 weeks, 6 months and 12 months

	BL	8 wks	6 mths	12 mths	F (df)	*p*-value	Change at 12 mths Mean (95% CI)	Effect size (d)
**WOMAC**								
Pain (0 to 20)	7.91 (0.46)	6.57 (0.36)	6.30 (0.39)	5.95 (0.46)	11.01_(3,201)_	< 0.001	1.95 (1.1 to 2.7)	0.5
Stiffness (0 to 8)	3.88 (0.21)	3.16 (0.19)	3.19 (0.17)	3.13 (0.19)	6.19_(3,201)_	< 0.001	0.75 (0.3 to 1.1)	0.4
Function (0 to 68)	24.98 (1.41)	20.66 (1.28)	19.86 (1.37)	19.63 (1.44)	10.02_(3,201)_	< 0.001	5.35 (2.8 to 7.8)	0.4
Total (0 to 96)	36.77 (1.94)	30.29 (1.66)	29.39 (1.84)	28.72 (2.00)	12.25_(3,201)_	< 0.001	8.06 (4.6 to 11.4)	0.5

Values are mean (SE). Lower scores indicate improvement; F-values and p-values are for repeated measures ANOVA. Change at 12 months values are mean (95% CI). Effect size is for pair wise comparison between baseline and 12 month scores.

**Table 3:** SF-36 scores at baseline, 8 weeks, 6 months and 12 months

**SF-36 **(0 to 100)	**BL**	**8 wks**	**6 mths**	**12 mths**	**F (df)**	***p*-value**	**Change at 12 mths Mean (95% CI)**	**Effect size (d)**
Physical Function	47.36 (2.49)	52.00 (2.62)	56.13 (2.62)	55.66 (3.13)	5.47_(3,201)_	< 0.001	7.73 (2.70 to 12.70)	0.3
Role Physical	29.77 (4.54)	41.54 (4.87)	43.01 (4.99)	46.69 (5.23)	4.48_(3,201)_	0.004	16.91 (7.20 to 26.50)	0.4
Bodily Pain	35.50 (1.85)	40.39 (2.06)	41.52 (2.52)	43.64 (2.49)	3.49_(3,201)_	0.017	8.14 (3.03 to 13.20)	0.5
General Health	63.72 (2.58)	67.05 (2.25)	69.13 (2.57)	69.79 (2.50)	3.42_(3,201)_	0.018	6.07 (1.50 to 10.50)	0.3
Vitality	50.41 (2.73)	56.38 (2.10)	57.30 (2.41)	60.73 (2.58)	7.64_(3,201)_	< 0.001	10.30 (5.20 to 15.30)	0.4
Social Function	69.64 (3.04)	78.67 (2.46)	76.72 (2.81)	79.98 (2.72)	5.16_(3,201)_	0.002	10.30 (4.80 to 15.70)	0.4
Role Emotional	53.97 (5.31)	66.14 (4.81)	71.05 (4.98)	73.01 (4.80)	5.27_(3,201)_	0.002	19.04 (9.10 to 28.90)	0.4
Mental Health	71.02 (2.25)	77.11 (1.70)	79.00 (1.90)	78.73 (2.11)	9.34_(3,201)_	< 0.001	7.70 (3.60 to 11.70)	0.4

Values are mean (SE). Higher scores indicate improvement; F-values and p-values are for repeated measures ANOVA. Change at 12 months values are mean (95% CI). Effect size is for pair wise comparison between baseline and 12 month scores.

During the 6-week intervention, pain levels (VAS) decreased significantly from a mean (SE) 5.61(2.3) to 4.1(2.6) (baseline to week 8).

At baseline knee flexion range of motion was 117 degrees (range 70 to 145 degrees) and extension range of motion was 180 degrees (range 165 to 195 degrees). At the 12 month follow-up flexion range of motion had increased 4 degrees (95% CI 0 to 8 degrees). Mean change in extension range of motion was 2 degrees (95%CI 0 to 3.8 degrees).

At baseline 47% of participants were able to achieve the target time of 10 seconds single leg balance thus creating a ceiling effect, therefore no improvement could be obtained. This proportion was unchanged at the completion of the study. The other participants who were unable to balance for 10 seconds at baseline (and could potentially improve) achieved a mean of 3 seconds for both legs with a mean improvement of 3 seconds (95%CI right leg 2.1 to 4.3 seconds; left leg 1.9 to 4.5 seconds).

## Discussion

This SM program differs from others as the intervention is specific not only to their pathology (OA), but also to the joint affected (knee). Health professionals use their expertise to deliver information and education covering a wide spectrum of topics, while utilising the constructs of SM to enable participants to take control of their OA and to improve their self-efficacy.

The outcomes of this clinical intervention were a decrease in pain, improvement in quality of life, and an improvement in OA specific health status. These findings have a number of important implications for the management of patients with OA.

Patients with OA of the knee have identified pain and problems with daily activities as the most important problems associated with their condition [25]; hence the results of this study are well matched to their priorities. Moreover, the aim of this multidisciplinary SM program, to empower people to manage their condition [1], is consistent with the preference of patients to actively manage their own condition [25], and this approach is likely to have benefits in terms of both disease and financial outcomes.

Although SM in chronic illness has been studied extensively, most arthritis programs have been designed to be delivered by lay facilitators and are generic in their focus. This study targeted a specific site – the knee, and a single pathology (OA), while using health professionals to provide disease education, exercise advice in keeping with principles of joint protection, healthy life style options, and relevant information within the self-management construct to achieve these positive short and medium term results. As arthritis SM programs designed to be delivered by health professional leaders have rarely been conducted or evaluated, the results of this case series project are likely to be important in the future planning of SM programs.

We suggest that the improvements demonstrated in this study may be a result of a number of different factors. HP's provided modelling potential [26] with the orientation towards skills and expertise as well as support rather than a support and empathy orientated framework offered by lay leaders.

The delivery of specific information, education and direction in an easily digestible format allowed participants to understand the rational behind the theory included in the program [6]. Understanding the reason for the adoption of concepts in the program allowed participants to be self-motivated to change behaviour and therefore be more compliant long term [27]. An example of this is exercise. As participants increased their exercise level over the 8-week intervention period, most had a reduction of pain, improved wellbeing and feelings of accomplishment that motivated them to continue. What was previously negative reinforcement (pain) changed to become positive reinforcement (less pain and improved well being) [28].

Education in the correct use of medication and analgesia is linked to the point above. Fear of pain is often a greater limiter than pain itself – hence the fear of developing pain will inhibit people from attempting certain activities. Most people attending this QA program did not take analgesia to adequately control their pain. When participants felt confident that they could control their pain, they became more confident that aspect of their OA was manageable (and would exercise more, for example) [4]. Cognitive pain management was also part of the program syllabus and complemented pharmacological pain management.

Developing problem solving skills was encouraged. HP's skilled in musculoskeletal conditions offered advice or alternatives when hurdles were encountered so that participants achieved solutions rather than giving up, thereby improving SMART goal success and consequently improving self-efficacy [26]. Subsequent problems encountered were more likely to be problem solved rather than met with a defeatist attitude [6].

Using a self-management format to embrace HP skills, expertise and knowledge to deliver education in a format that participants could relate to in everyday life was hoped to improve self-efficacy in areas across the OA spectrum. It was thought that this would promote healthy life style and behaviour changes that would improve pain, physical function and quality of life.

### Pain

In this study pain was measured in a number of ways, all demonstrating an improvement. A number of aspects of the self-management intervention may have contributed to the reduced pain levels reported by participants. Both aerobic and resistance exercise in a home-based exercise program have been shown to significantly reduce knee pain in-patients with OA [29,30]. An important component of the OAK intervention is discussion on the formulation of a comprehensive home exercise program that incorporates strengthening, endurance, balance and flexibility components. Participants were not taken into a gym or given individual personal training; however they were encouraged to pursue that option independently.

The exercise component was not controlled and participants freely chose the type of exercise/s and the degree to which they would comply. By providing a number of exercise alternatives, it was hoped that exercise routines would become habitual by the end of the 6-week program. In accordance with self-management principles, participants were motivated to use their "library" of exercise choices when planning their weekly goals. The use of goal setting with participants promoted good adherence to the exercise program, as reported each week, but data regarding adherence were not collected for this study.

As well as exercise instruction and cognitive therapies, medication usage and therapeutic dosing principles in particular for analgesia were taught to encourage medication compliance and effective pain management. The average age of participants was 66 years and most had several co-morbidities requiring medication (Table 1). Many participants had an aversion to medications and delayed taking analgesia until their pain was acute and therefore more difficult to control. Pain management guidelines were discussed with the aim of determining patterns of pain. For example short term "around the clock" analgesia dosing for acute pain, or "as needed" analgesia for intermittent pain.

It is likely that the OAK intervention has facilitated better pain-coping skills that are important predictors of disability associated with OA. Previous studies have reported that catastrophizing and negative self-statements are associated with increased knee pain [31]. In the OAK intervention, participants were taught strategies for cognitive symptom management such as distraction, guided imagery, relaxation and thought challenging techniques that are considered to be important additional measures of pain management in people with OA [30,32].

### Health Status

Participants reported considerable improvements in physical function. Like pain, functional improvements were reflected by changes in a number of the parameters measured. It is generally accepted that the WOMAC questionnaire has greater specificity and consequently better responsiveness for people with OA [33], nonetheless, the SF-36 also reflected these changes.

Interpreting these results requires some understanding of the value patients place on improvements of this magnitude. Establishing this can be difficult. A number of methods, each with strengths and limitations, have been used but findings are not entirely consistent. Improvements of 9% to 10% in WOMAC scores in response to rofecoxib or ibuprofen were perceptible to patients with OA knee [34] when anchored against a patient global assessment of response to therapy. Changes observed in our study were generally more than twice this magnitude. On the other hand the 21.6% improvement in WOMAC function was somewhat less than 26%, the minimal level suggested by Tubach et al [35] as clinically important.

Expressed as effect sizes in standard deviation units the improvements in the WOMAC pain and SF-36 bodily pain domains would be considered moderate [23]. The consistency of this effect between different outcome tools supports the validity of the change. Effect sizes for the WOMAC functional domain and for the SF-36 mental health domains were slightly lower at 0.4. Notably these effect sizes are larger or comparable to the pooled effect sizes for general pain from systematic reviews of NSAID therapy [36] and aerobic walking [37] (0.33 and 0.52 respectively), although larger effects are often observed in uncontrolled studies. Further context for interpretation of the improvements we observed in quality of life measured by the SF-36 may be provided by considering the average decline of 2.1 points over 12 months reported in people with OA in this age group [15].

### Limitations

The subjects who attended this quality assurance program were typical of those who attend self-management programs run by Arthritis WA. Over representation in the highest socio-economic group (Table 1) may affect the reproducibility of this program, however, the demographics of the area this program was conducted in are consistent with this attendance statistic. These results should be interpreted with caution as this limits the generalization to other socio-economic groups. Testing the OAK program with other socio-economic groups was outside the limitations of this QA program.

It is important to note that no control period or control group were available for comparison. Consequently, the clinical improvements observed in this cohort should be interpreted with caution. Despite this, improvements in response to this disease specific self-management program delivered by health professionals are encouraging and have interest. We therefore propose to further evaluate the benefits of this program using a more rigorous study design.

## Conclusion

Improvements in pain, health status and physical function were observed in response to our SM education program specifically designed for people with OA knee, delivered by health professionals. Health professionals providing the program enabled inclusion of disease specific content, not found in other arthritis SM programs, to be incorporated in the OAK program. The long-term gains demonstrated in OAK are not reflected in other arthritis SM programs. Furthermore rigorous investigation of the benefits of this approach to treatment is warranted.

## Abbreviations

SM: self-management; OAK: osteoarthritis of the knee; OA: osteoarthritis; HP: health professional; QA: quality assurance; WOMAC: Western Ontario and McMaster Universities Osteoarthritis Index; VAS: visual analogue scale; CI: confidence interval; ASMP: Arthritis Self-Management Program; SF-36: Medical Outcomes Short Form 36 Questionnaire; SMART: **s**pecific, measurable, achievable, realistic, timely; NSAID: non-steroidal antiinflammatory drug

## Competing interests

The authors declare that they have no competing interests.

## Authors' contributions

SC, RP, GC, JM contributed to study design. SC and HC were responsible for the acquisition of data and SC the data-entry. All authors contributed to analysis and interpretation of the data. KB and SC contributed to manuscript preparation. All authors have approved the final version of the manuscript.

## Pre-publication history

The pre-publication history for this paper can be accessed here:


